# Incidence trends of lung and gastroenteropancreatic neuroendocrine neoplasms in Switzerland

**DOI:** 10.1002/cam4.3524

**Published:** 2020-10-20

**Authors:** Heba Alwan, Stefano La Rosa, Peter Andreas Kopp, Simon Germann, Manuela Maspoli‐Conconi, Christine Sempoux, Jean‐Luc Bulliard

**Affiliations:** ^1^ Vaud Cancer Registry Centre for Primary Care and Public Health (Unisanté) University of Lausanne Lausanne Switzerland; ^2^ Institute of Pathology University Hospital and University of Lausanne Lausanne Switzerland; ^3^ Division of Endocrinology Diabetology and Metabolism University Hospital and University of Lausanne Lausanne Switzerland; ^4^ Division of Endocrinology Metabolism and Molecular Medicine Northwestern University Chicago IL USA; ^5^ Neuchâtel and Jura Cancer Registry Neuchâtel Switzerland

**Keywords:** epidemiology, incidence, neuroendocrine neoplasms, Switzerland, trend

## Abstract

The incidence of neuroendocrine neoplasms (NENs) seems to increase worldwide. Long‐term, population‐based series that consider tumor differentiation are, however, sparse. We assessed the incidence trend of lung and gastroenteropancreatic (GEP) NENs according to the latest International Agency for Research on Cancer/World Health Organization classification over a 41‐year time period in two Swiss regions. All cases of lung and GEP NENs recorded in the Vaud and Neuchâtel Cancer Registries from 1976 to 2016 were included. NENs were stratified into well‐differentiated neuroendocrine tumors (NETs) and poorly differentiated neuroendocrine carcinomas (NECs). Changes in annual age‐standardized incidence rates were calculated for lung and GEP NETs and NECs by sex. Of 4,141 patients diagnosed with NENs, 65% were men. The incidence of lung NETs among men and women increased by 3.9%/year (95% CI: −5.3, 14.1%) and 4.9%/year (0.1, 9.9%), respectively, between 1976 and 2016. The incidence of lung NECs decreased by 2.6%/year (−3.1,‐1.8%) in men from 1985 to 2016 whereas it increased in women between 1976 and 1998 by 6%/year (4.2, 7.9%). For GEP NETs, a steady annual increase in incidence occurred between 1976 and 2016 with a magnitude of 1.7% (0.7, 2.7%) in men and 1.3% (0.5, 2.1%) in women. No significant trend in incidence of GEP NECs was found for both sexes. The incidence trends of lung NECs in men and women parallel changes in smoking prevalence in the population. Causes of the increase in incidence of GEP NETs are likely multifactorial. Our study supports the importance of evaluating the epidemiology of NENs by tumor differentiation.

## Graphical abstract

Over the last four decades, the incidence trends of neuroendocrine lung neoplasms (NENs) parallel, in men and women, changes in smoking prevalence in the population. The steady increase in gastroenteropancreatic (GEP) NENs in both sexes is likely multifactorial but has not yet been fully elucidated.

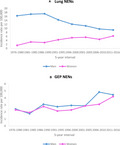

## 1 INTRODUCTION

Neuroendocrine neoplasms (NENs) constitute a heterogeneous group of neoplasms that can virtually arise in any organ of the body, but are most commonly found in the gastroenteropancreatic (GEP) tract and the lungs.^1^, ^2^, ^3^ NENs were first identified over a century ago by Siegfried Oberndorfer who introduced the term “Karzinoid” or “carcinoma‐like” to describe a slow‐growing small bowel tumor.^2^ However, it became apparent that NENs can have a malignant behavior and therefore the term carcinoid is no longer recommended when referring to NENs.^2^ NENs are characterized by having neurosecretory granules^4^ and can secrete different hormones, which are frequently dependent on tumor site.

Small bowel NENs most often secrete serotonin and kallikrein, which can give rise to a characteristic paraneoplastic syndrome referred to as the “carcinoid syndrome.”^4^, ^5^, ^6^ In the pancreas, tumors may secrete pancreatic hormones or gastrin, a hormone that is frequently secreted by duodenal NENs. However, the carcinoid syndrome and the specific endocrine syndromes related to the secretion of the above‐mentioned hormones (glucagonomas, somatostatinomas, Zollinger‐Ellison syndrome, among others) are only present in a minority of cases and the vast majority of NENs are diagnosed because of bowel obstructions, pulmonary symptoms, local growth, metastatic disease, or as incidental findings.^5^, ^7^, ^8^

Over the years, the numerous and changing nomenclature and grading systems have led to confusion among clinicians and pathologists.^2^ Changes in the classification systems of NENs are summarized in Table 1. In 2018, the World Health Organization (WHO) and the International Agency for Research on Cancer (IARC) proposed unifying nomenclatures to classify NENs into well‐differentiated NENs, or neuroendocrine tumors (NETs), and poorly differentiated NENs, or neuroendocrine carcinomas (NECs).^4^ This classification, officially adopted in the last WHO classification of digestive NENs,^9^ has clinical utility as it can help better define tumor prognosis and guide management.^2^ NETs can be further classified into grades 1‐3 using three parameters: mitotic count, the Ki‐67 cell labeling index for GEP NETs, and in the lung, where the term carcinoid is still accepted, the presence or absence of necrosis.^4^

**Table 1:** World Health Organization (WHO) classifications of lung and gastroenteropancreatic neuroendocrine neoplasms

	Lung			Gastroenteropancreatic system							
	WHO 2015			WHO 2010				WHO 2019			
	Terminology	Mitotic index	Necrosis	Terminology	Mitotic index	Ki67 index		Terminology	Mitotic index		Ki67 index
Well‐differentiated^a^	TC	<2/2 mm^2^	no	NET G1	<2/10HPF		≤2%	NET G1		<2/2 mm^2^	<3%
	AC	2‐10/2 mm^2^	yes	NET G2	2‐20/10HPF		3‐20%	NET G2		2‐20/2 mm^2^	3‐20%
								NET G3	>20/2 mm^2^		>20%
Poorly differentiated^a^	NEC	>10/2 mm^2^	yes	NEC	>20/10HPF		>20%	NEC	>20/2 mm^2^		>20%
Mixed neoplasms	Combined	variable	variable	MANEC	variable		variable	MiNENs	variable		variable

Abbreviations: AC, atypical carcinoid; HPF, high power field; MANEC, mixed adenoneuroendocrine carcinoma; MiNEN, mixed neuroendocrine/non‐neuroendocrine neoplasm; NEC, neuroendocrine carcinoma; NET, neuroendocrine tumor; TC, typical carcinoid.

^a^ Morphologically well‐differentiated or poorly differentiated neoplasm.

The incidence of NENs seems to increase worldwide but the magnitude of the trend varies widely across studies.^1^, ^19^ This is likely due to the rarity and heterogeneity of these tumors, differences in data sources used (hospital‐based vs. population‐based series), changes in the classification and nomenclature used over time, as well as variability in ascertainment.^12^ For example, in the United States, the age‐adjusted incidence rate for NENs increased more than sixfold between 1973 and 2012 (from 1.1 to 7 per 100,000),^11^ while in the Netherlands a doubling of the incidence has been observed between 1990 and 2010 (from 2.1 to 4.9 per 100,000)^13^.

Despite the increasing number of publications on NENs in recent years, most studies could not assess the incidence of NENs from a population‐based perspective, did not report incidence rates by tumor differentiation or histological grade, and were conducted over a limited time period. Therefore, the current study aims at evaluating the incidence trend of lung and GEP NENs according to the most recent WHO/IARC classification over a 41‐year time period in two Swiss populations, based on long‐established and high‐quality cancer registry data.^20^

## 2 MATERIALS AND METHODS

Data for this study were extracted from the population‐based Vaud and Neuchâtel Cancer Registries which collect, document, and record all new cancer cases diagnosed among their resident populations since 1974 (total population in 2016: 951,514).^21^ Ascertainment of most cases is derived from multiple sources and cancer registration has a high level of completeness.^20^ Recorded data include demographic characteristics, primary tumor site, and morphology according to the International Classification of Diseases for Oncology (ICD‐O‐1 to ICD‐O‐3). All cases of lung (ICD‐O topography codes: C34.0 ‐ C34.9) and GEP (ICD‐O topography codes: C15.0 ‐ C25.9) NENs diagnosed over the 41‐year period 1976‐2016 were included in our analyses.

Tumor grade was available in 24% of cases because reporting proliferation indexes of tumors were only recently introduced routinely. Instead, we used the WHO/IARC recommendations to divide NENs into well‐differentiated NETs and poorly differentiated NECs.^4^ Cases registered as typical carcinoid tumors, enterochromaffin cell and enterochromaffin‐like cell carcinoid tumors, tubular carcinoids, atypical carcinoids, islet cell carcinomas, insulinomas, glucagonomas, gastrinomas, vipomas, somatostatinomas, and enteroglucagnomas were classified as NETs (ICD‐O‐3: 8240‐42, 8245, 8249, 8150‐53, and 8155‐57). Large and small cell NECs, and NECs not otherwise specified (NOS) were classified as NECs (ICD‐O‐3: 8013, 8041‐44, and 8246). Goblet cell carcinoids (ICD‐O‐3: 8243) were not included as they are now considered low‐grade adenocarcinomas according to the 2019 WHO classification for digestive tumors.^22^ To circumvent changes in morphological classifications over time, we re‐categorized NENs according to the 2013 ICD‐O‐3.1 classification, even for tumors diagnosed before this date. NENs previously diagnosed as grade 1 NECs and as grade 2 NECs were thus recoded as 8240 (rather than 8246) and as 8249 (rather than 8246), respectively.

Cases were analyzed by sex, age and year of diagnosis, tumor type (NET vs. NEC), and tumor site (lung vs. GEP). Age‐standardized incidence rates per 100,000 per year were calculated using the 1976 European standard population. We fitted a log‐linear regression model to estimate the annual percent change in the standardized rates, with calendar year as the predictor variable. Joinpoint statistical software (version 4.3; Surveillance Research Program, National Cancer Institute, Bethesda, MD) was used to estimate the model parameters, and to provide their standard errors allowing for heteroscedasticity management and calculation of confidence intervals for annual percent change in age‐standardized incidence rates. For GEP NECs, trend analyses were restricted to the time period 1996‐2016 for men and 1998‐2016 for women due to an insufficient number of cases before these years.

## 3 RESULTS

There were 4141 patients diagnosed with NENs in these two Swiss regions between 1976 and 2016, of which 65% were men. The median age at diagnosis was 65 years (interquartile range: 57‐73) for men and 64 years for women (interquartile range 52–73). Table 2 presents the distribution of the 3041 lung and 1100 gastroenteropancreatic NENs by morphological type. Most lung NETs were typical carcinoids (91% of cases). In the lungs, 95% of NECs were small cell lung carcinomas (SCLC), whereas 62% of GEP NECs were registered as NEC, NOS.

**Table 2:** Distribution of lung and gastroenteropancreatic neuroendocrine neoplasms between 1976 and 2016 in two Swiss regions by tumor type.

	Lung NENs		GEP NENs		Total	
	Distribution		Distribution		Distribution	
Tumor type	n	%	n	%	n	%
NET, total	225	7.4	962	87.5	1187	28.7
Carcinoid tumor	204	90.7	827	86.0	1031	86.9
Enterochromaffin cell carcinoid	2	0.9	27	2.8	29	2.4
Enterochromaffin‐like cell carcinoid	1	0.4	10	1.0	11	0.9
Tubular carcinoid	‐	‐	5	0.5	5	0.4
Atypical carcinoid	18	8.0	46	4.8	64	5.4
Islet cell carcinoma	‐	‐	6	0.6	6	0.5
Insulinoma	‐	‐	25	2.6	25	2.1
Glucagonoma	‐	‐	7	0.7	7	0.6
Gastrinoma	‐	‐	8	0.8	8	0.7
Somatostatinoma	‐	‐	1	0.1	1	0.1
NEC, total	2816	92.6	138	12.6	2954	71.3
Small cell carcinoma	2670	94.8	41	29.7	2711	91.8
Large cell carcinoma	90	3.2	11	8.0	101	3.4
NEC, NOS	56	2	86	62.3	142	4.8

Abbreviations: GEP, gastroenteropancreatic; NEC, neuroendocrine carcinomas; NEN, neuroendocrine neoplasms; NET, neuroendocrine tumors; NOS, not otherwise specified.

**Figure 1:**
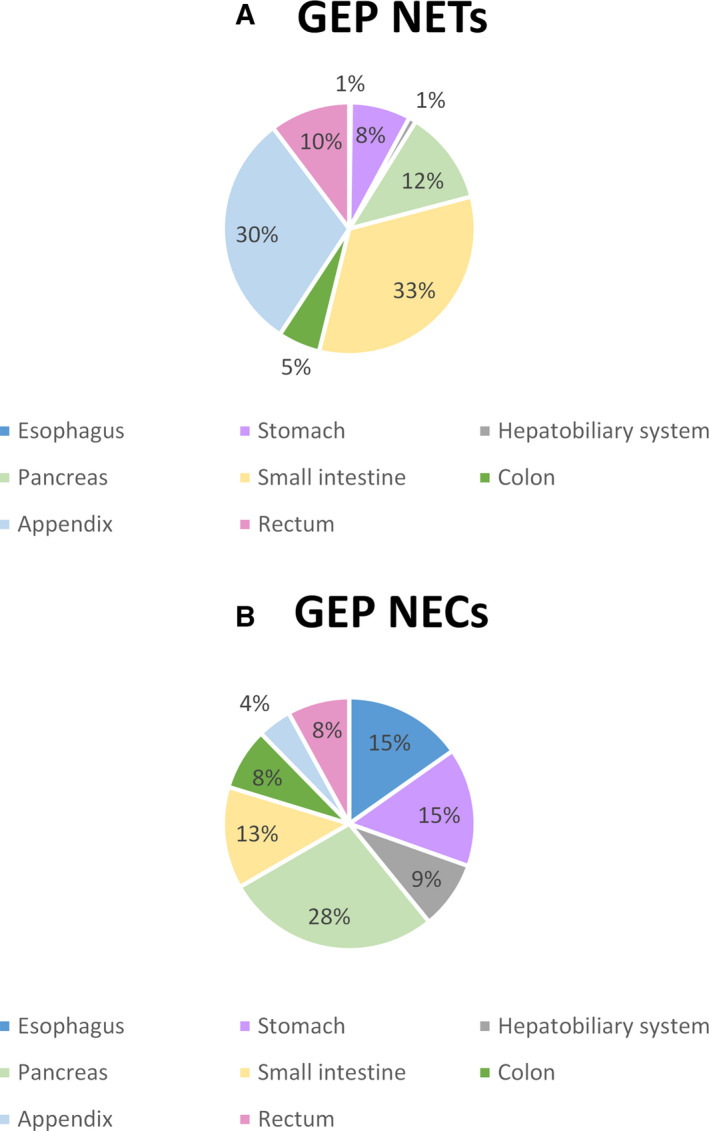
Anatomic distribution (%) of gastroenteropancreatic (GEP) neuroendocrine tumors (NET) tumors (A) and GEP neuroendocrine carcinomas (NEC) tumors (B) by site

There was a slight female preponderance in NET cases (54% and 51% for lung and GEP, respectively), whereas NECs patients were more often men (72% and 58% for lung and GEP NECs, respectively). The majority of GEP NETs were located in the small intestine (33%) or the appendix (30%) (Figure 1 **).** The most common site for GEP NECs was the pancreas (28%).

**Figure 2:**
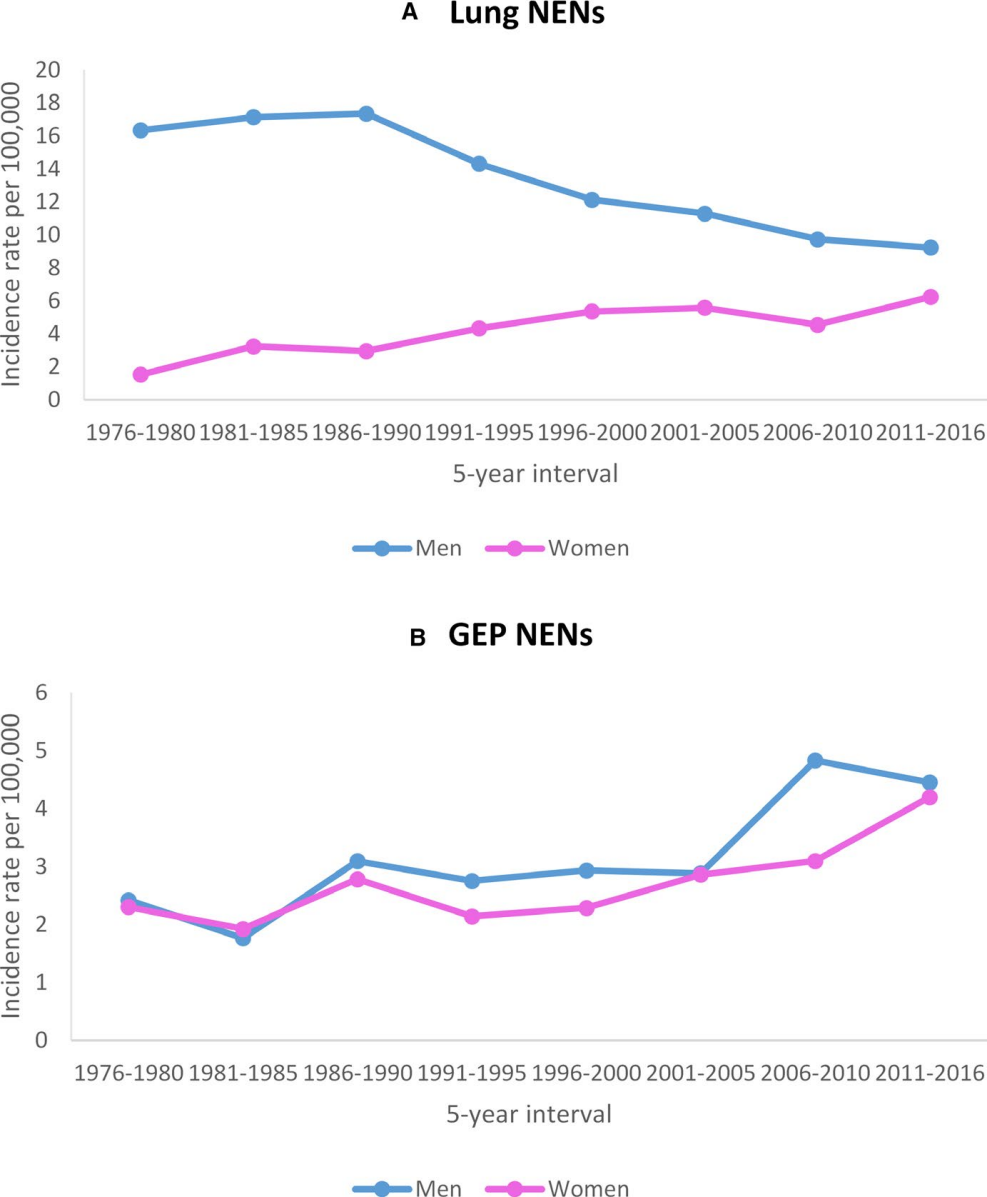
Age‐standardized incidence rates of neuroendocrine neoplasms (NENs) by 5‐year intervals and sex in the lung (A) and gastroenteropancreatic (GEP) tract (B). Rates are per 100,000 and age‐adjusted to the 1976 European standard population.

Figure 2 displays the age‐standardized incidence rates of lung and GEP NENs by sex and quinquennial time period. Among men, the incidence of lung NENs decreased from 16.3 cases per 100,000 in 1976–1980 to 9.2 cases per 100,000 in 2011–2016, whereas the opposite trend was observed among women (increase from 1.5 cases per 100,000 in 1976–1980 to 6.2 cases per 100,000 in 2011–2016). However, the incidence rate of lung NENs remained higher in men than in women. For GEP NENs, there was an upward trend in both sexes (from 2.4 cases per 100,000 in 1976–1980 to 4.5 cases per 100,000 in 2011–2016 among men and from 2.3 cases per 100,000 in 1976–1980 to 4.2 cases per 100,000 in 2011–2016 among women).

The age‐standardized incidence rates of lung and GEP NENs by sex, 5‐year time period, and tumor type (NET vs. NEC) are reported in Table 3.

**Table 3:** Age‐standardized incidence rates of neuroendocrine neoplasms by 5‐year intervals, sex, and tumor type in the lung and gastroenteropancreatic (GEP) tract by tumor type. Rates are per 100,000 and age‐adjusted to the 1976 European standard population

Incidence year interval	Men				Women			
	Lung		GEP		Lung		GEP	
	NET	NEC	NET	NEC	NET	NEC	NET	NEC
1976–1980	0.60	15.73	2.26	0.16	0.39	1.14	2.27	0.03
1981–1985	0.48	16.66	1.60	0.17	0.64	2.60	1.87	0.05
1986–1990	0.26	17.09	2.96	0.13	0.47	2.48	2.78	0.00
1991–1995	0.63	13.69	2.65	0.11	0.42	3.92	2.12	0.02
1996–2000	1.27	10.86	2.36	0.58	0.59	4.77	2.05	0.24
2001–2005	0.45	10.84	2.32	0.56	0.37	5.21	2.41	0.46
2006–2010	0.46	9.27	4.21	0.62	0.71	4.70	3.10	0.60
2011–2016	0.74	8.50	3.55	0.90	1.19	5.05	3.70	0.50

Abbreviations: GEP, gastroenteropancreatic; NET, neuroendocrine tumors; NEC, neuroendocrine carcinomas.

The trends in incidence rates from 1976 to 2016 of lung and GEP NENs by sex and tumor type are displayed in Table 4. The incidence of lung NENs among men increased non significantly by 4.9% (95% confidence interval (CI): −3 to 13.4) per year between 1976 and 1981 and gradually decreased thereafter by 2.2% per year (95% CI: −2.7 to −1.8). In contrast, the incidence of lung NENs in women increased between 1976 and 1998 by 5% per year (95% CI: 3.1 to 6.9), before stabilizing. For GEP NENs, a steady annual increase in incidence occurred between 1976 and 2016 with a magnitude of 2.2% in men (95% CI: 1.4 to 3.1) and 1.8% in women (95% CI: 1.0 to 2.7).

**Table 4:** Trends in the incidence rate of neuroendocrine neoplasms from 1976 to 2016.

NEN category	Trend 1			Trend 2		
	Years	EAPC (%)	95% CI (%)	Years	EAPC (%)	95% CI (%)
Lung NENs						
Men	1976‐1981	4.9	−3 to 13.4	1981‐2016	−2.2	−2.7 to −1.8
Women	1976‐1998	5	3.1 to 6.9	1998‐2016	0.7	−0.8 to 2.2
GEP NENs						
Men	1976‐2016	2.2	1.4 to 3.1			
Women	1976‐2016	1.8	1.0 to 2.7			
Lung NETs						
Men	1976‐2016	3.9	−5.3 to 14.1			
Women	1976‐2016	4.9	0.1 to 9.9			
Lung NECs						
Men	1976‐1985	1.3	−2.1 to 4.9	1985‐2016	−2.6	−3.1 to −2
Women	1976‐1998	6	4.2 to 7.9	1998‐2016	0	−1.4 to 1.4
GEP NETs						
Men	1976‐2016	1.7	0.7 to 2.7			
Women	1976‐2016	1.3	0.5 to 2.1			
GEP NECs						
Men	1996‐2016	6.7	−6.7 to 22			
Women	1998‐2016	5.4	−12.1 to 26.4			

Abbreviations: EAPC: estimated annual percentage change; GEP: gastroenteropancreatic; NEC: neuroendocrine carcinomas; NEN: neuroendocrine neoplasms; NET: neuroendocrine tumors.

The incidence of lung NETs (carcinoids) did not reveal any significant trend in men, but increased in women by 4.9% (95% CI: 0.1 to 9.9) per year between 1976 and 2016. For lung NECs, a decrease in the incidence of 2.6% per year has been observed in men (95% CI: −3.1 to −2) between 1985 and 2016 whereas the incidence has increased in women between 1976 and 1998 by 6% per year (95% CI: 4.2 to 7.9), after which it stabilized. For GEP NETs, the incidence has moderately but significantly increased in both men and women between 1976 and 2016 at a rate of 1.7% per annum in men (95% CI: 0.7 to 2.7) and 1.3% per annum in women (95% CI: 0.5 to 2.1). GEP NECs did not show an incidence trend for both sexes.

## 4 DISCUSSION

In this population‐based study from two Swiss cancer registries, we assessed the incidence trend of lung and GEP NENs over four decades using the most recent WHO/IARC classification. We observed a decrease in incidence of lung NENs in men between 1981 and 2016 mostly explained by the downward trend in lung NECs. Among women, the incidence of lung NENs increased from 1976 to 1998 driven by a rise in both lung NETs and NECs. This was followed by a stabilization in incidence in lung NENs mainly due to a stable incidence of lung NECs. Incidence of GEP NENs has steadily increased by the same magnitude in both men and women between 1976 and 2016 due to an increase in GEP NETs.

Only few studies have analyzed the incidence trend of NENs according to tumor differentiation.^13^, ^15^, ^23^ It is noteworthy that NETs and NECs are two distinct disease entities in terms of histological characteristics, and they have a different molecular background, tumor biology, prognosis, and treatment.^2^, ^4^ Moreover, NETs predominate at some sites (e.g., in the small intestine) whereas NECs constitute the majority of NENs in other organs.^4^ This is consistent with our findings that most GEP NENs were NETs, whereas the majority of lung NENs were NECs.

In regards to morphology, the vast majority of lung NETs (carcinoids) were typical carcinoids, consistent with the literature.^24^ We observed a significant increase of 5% per year between 1976 and 2016 in lung NET incidence in women and of 4.9% per year in men (though statistically non‐significant). Previous studies have also shown an increase in lung NETs over time,^1^, ^11^, ^15^, ^25^ although a sex difference in incidence trends for lung NETs is rarely reported. Leoncini et al. found a higher trend in incidence rate of lung NETs among women versus men in the Surveillance, Epidemiology, and End Results (SEER) program in the Unites States.^15^ Conversely, in the Netherlands a decreasing incidence of lung NETs (carcinoids) in men over time and a stable incidence in women has been reported.^13^ As risk factors for lung NETs (carcinoids) are still largely unidentified, it remains uncertain whether this increase in incidence could be due to changes in exposure to putative risk factors. In particular, it remains unclear whether smoking is associated with lung NETs (carcinoids).^13^ It has also been postulated that the increase in imaging procedures might be responsible for the observed increase in lung NETs (carcinoids).^11^

Concerning the incidence trend of lung NECs, we observed a decrease of 2.6% per year in men from 1985 to 2016 and a strong increase of 6% per year in women from 1976 to 1998, followed by a plateau. The majority of our lung NECs were SCLC, which is a cancer strongly associated with smoking.^26^ Large cell lung cancer is also associated with smoking^27^. Studies focusing on NENs often exclude SCLC based on their different biological and histological properties as compared to other NENs. Studies that included SCLC also found that SCLC was the most common lung NEC and reported sex‐specific trends in incidence similar to our findings.^13^, ^15^, ^23^, ^25^, ^26^ These trends are consistent with smoking habits as the prevalence of tobacco use among women lags behind that of men by a few decades.^28^ This can explain the upward incidence trend of lung NECs among women up to 1998, whereas the incidence in men has been decreasing since around 1985 in the two Swiss regions covered in our analysis.

Our finding that the incidence of GEP NETs has been steadily increasing for over four decades concurs with the literature.^13^, ^15^ In line with previous studies, most GEP NETs were located in the small intestine and the appendix.^13^, ^17^, ^23^ An earlier study in the Vaud population also reported an increase in GEP NETs incidence between 1974 and 1997 mainly among men.^16^ Although an increase in incidence of GEP NECs is apparent over time, this trend did not reach statistical significance. Studies that have reported incidence trends of GEP NENs by level of differentiation (NETs vs. NECs) have shown an upward trend in the incidence of GEP NECs.^13^, ^15^ Our study might be underpowered to detect a significant change in incidence due to the relatively small number of GEP NEC cases.

Whether the increase in incidence in GEP NENs reported in several international studies is true or spurious remains unclear. Some have attributed the increase in GEP NETs to the increased use of endoscopies and radiological procedures, and an increased medical sensitivity in regards to these neoplasms.^13^, ^23^ In Switzerland, the use of computed tomography scans has more than doubled between 1998 and 2008, which could explain the increased detection rate for NENs.^29^ This may be particularly true for small, noninvasive tumors that might not have been otherwise detected.^23^ This assumption is consistent with our results as the incidence of well‐differentiated GEP NETs rises, while that of poorly differentiated GEP NECs does not. In support of this assumption, a recent Canadian study has shown a rising incidence of NETs concomitant with a decreased incidence of metastatic NENs.^6^

Other possible contributory factors for the rising incidence of GEP NETs are the changes in both morphological and clinical classification systems, and an increased awareness of NENs among pathologists and clinicians.^13^, ^30^ For example, the term “well‐differentiated neuroendocrine carcinoma” introduced in the 2000 WHO classification to define metastatic well‐differentiated neuroendocrine neoplasm created some confusion.^31^ Indeed, it was intended to define a well‐differentiated tumor now categorized as NET. The term NEC is now only applicable to a poorly differentiated neuroendocrine neoplasm. Introduction of immunohistochemistry methods might have also contributed to this rising incidence as these methods facilitate the diagnosis of NEN cases for pathologists,^30^ although this should increase the incidence of all GEP NENs and not only of GEP NETs.

If the increase in GEP NENs were solely explained by a rise in incidental discoveries or increased awareness, then one would expect the incidence of all GEP NENs to increase in the same proportion.^12^ However, the reported magnitude of incidence trends of GEP NENs varies by organ.^12^ If there is a true rise in incidence of these neoplasms, the underlying factors and mechanisms remain to be elucidated. It has been suggested that the rising incidence of gastric NETs may be due to the increased use of proton pump inhibitors during the last decades.^12^

Our study has some limitations. We did not estimate the incidence of GEP NENs by organ due to the limited number of cases. Data on grade and stage were incomplete. To overcome this, we divided NENs into well‐differentiated and poorly differentiated neoplasms according to the latest WHO/IARC classification. Strengths of our study include its population‐based nature with a long follow‐up of 41 years and a high degree of registration completeness. Moreover, in contrast to most cancer registries that historically included only malignant tumors, the Vaud and Neuchâtel cancer registries have recorded all NENs since their establishment, despite the fact that some NEN types were initially considered as benign neoplasms. This strongly reduces the risk of bias due to changes in pathological classification criteria in incidence trend analyses.

In conclusion, the findings of this study support the importance of evaluating the epidemiology of NENs by their level of differentiation. The incidence trends of lung NECs in men and women parallel the changes in smoking prevalence in the population. Causes of the steady increase in incidence of GEP NENs over the last four decades are not fully understood and are likely multifactorial. Future studies should ideally use population‐based data, apply the latest WHO/IARC classifications for NENs, and have complete information on histological grade and stage in order to better identify some of the reasons underlying the changes in the incidence trends of NENs.

## CONFLICT OF INTEREST

The authors declare no conflict of interest.

## ETHICS APPROVAL

The study was approved by the Human Research Ethics Committee of the canton of Vaud (Project‐ID 2019‐01328).

## Data Availability

Data available on request due to privacy/ethical restrictions.
